# Case Report: Immune Checkpoint Inhibitor-Induced Exuberant Tumor Inflammation With Accelerated Clinical Deterioration in Metastatic Renal Cell Carcinoma

**DOI:** 10.3389/fonc.2021.679177

**Published:** 2021-04-29

**Authors:** Dharmesh Gopalakrishnan, Rohit K. Jain, Laurie Herbst, Marcus Sikorski, Silpa Mandava, Gissou Azabdaftari, Bo Xu, Charles LeVea, Kevin Robillard, Marc S. Ernstoff, Saby George

**Affiliations:** ^1^ Department of Medicine, Roswell Park Comprehensive Cancer Center, Buffalo, NY, United States; ^2^ Department of Genitourinary Oncology, Moffitt Cancer Center and Research Institute, Tampa, FL, United States; ^3^ Department of Pathology, Roswell Park Comprehensive Cancer Center, Buffalo, NY, United States; ^4^ ImmunoOncology Branch, Developmental Therapeutics Program, National Cancer Institute, Bethesda, MD, United States

**Keywords:** renal-cell carcinoma, immune checkpoint inhibitor, tumor inflammation, nivolumab, pseudoprogression

## Abstract

Immune checkpoint inhibitors (ICIs) have revolutionized cancer therapy. Nivolumab, an anti-PD-1 monoclonal antibody, markedly improved overall survival in advanced renal cell carcinoma (RCC). However, ICIs can rarely trigger massive inflammation, a phenomenon characterized by rapid acceleration in radiographic tumor growth, the mechanisms underlying which are largely unknown. We report three patients with metastatic RCC who experienced rapid radiographic progression and clinical deterioration following treatment with nivolumab. However, histological analysis revealed no viable cancer despite the evidence of radiological progression. Instead, extensive necrosis and lymphohistiocytic infiltration were noted, as described previously in patients with ICI-induced pseudoprogression. Based on these observations, we postulate that exuberant antitumor inflammatory responses may contribute to adverse clinical outcomes in some patients with ICI-induced radiographic progression. Prospective studies incorporating tumor biopsies may shed more light on this rare phenomenon.

## Background

Monoclonal antibodies against programmed cell death protein-1(PD-1) and other co-inhibitory immune checkpoints act by reinvigorating antitumor effector T-cell responses (1). Nivolumab, a fully human Ig4 anti-PD-1 antibody, was demonstrated to significantly improve overall survival compared to everolimus among patients with previously treated clear cell RCC (RCC) in CheckMate-025, a phase 3 randomized open-label trial (2). Immune checkpoint inhibitors (ICIs) can occasionally lead to atypical responses such as pseudoprogression and hyperprogression. Pseudoprogression is defined as a transient radiological worsening followed by shrinkage of tumors with continued therapy (3). This phenomenon partly explains the benefit from treatment beyond radiological progression in patients who do not experience overt clinical deterioration (4, 5). A small minority of patients experience a more dramatic acceleration in tumor growth along with rapid clinical deterioration when exposed to ICIs, a phenomenon termed hyperprogression (3, 6). The mechanisms underlying ICI-related hyperprogressive disease remain poorly defined. We describe three patients with metastatic RCC who experienced rapid radiographic progression on nivolumab and analyze their tumor histologies. This study was approved by our Institutional Review Board (BDR No. 120019) and Informed consent was obtained from the next of kin.

## Case Presentation

**Figure 1:**
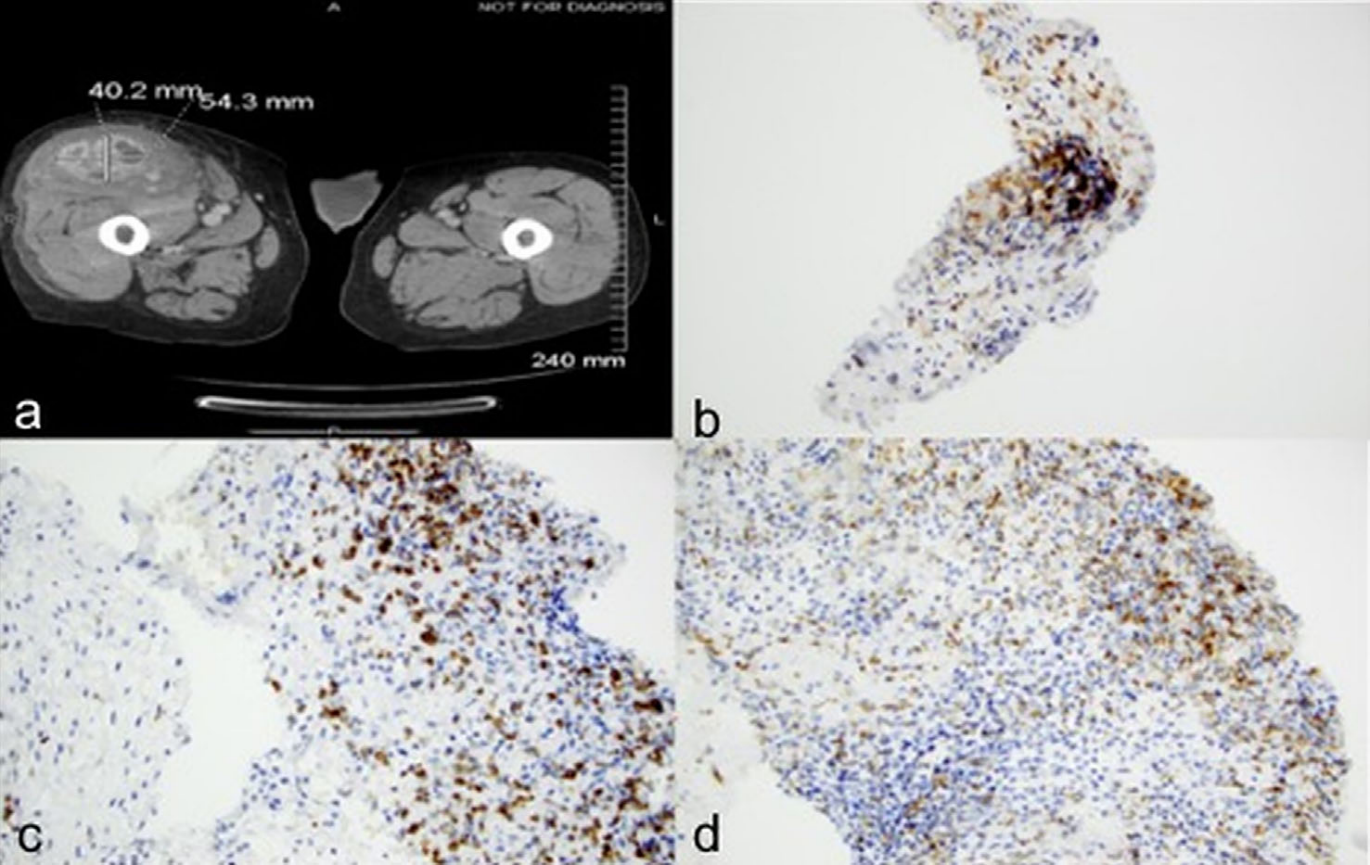
Case 1 demonstrating **(A)** Increase in size of right thigh mass **(B)** CD3 positive **(C)** CD5 positive **(D)** CD 68 positive immunohistochemical staining.

Case 1: A 68-year-old male who underwent left radical nephrectomy for a 10.5 cm pT3aNx, Fuhrman grade 2, clear cell RCC, developed radiographic recurrence in the nephrectomy bed a year later. Over the next 4.5 years, he was treated sequentially with sunitinib, axitinib, everolimus, and pazopanib. He also underwent metastasectomy with excision of retroperitoneal masses, resection of the diaphragm, and splenectomy. He also received palliative radiation after T10-T11 laminectomy for epidural metastasis with spinal cord compression. He was eventually started on nivolumab 3mg/kg every 2 weeks. Staging scans at this time showed lung, liver, omental and skeletal metastases. Also noted was a small anterior right thigh intramuscular lesion which had been stable for several preceding months. Follow-up CT scans after 6 doses of nivolumab showed marked enlargement of the right thigh intramuscular mass to 4.0 x 5.4 cm with contrast enhancement (**Figure 1A**), but stable disease in the lungs, liver, bones, omentum, and the nephrectomy bed. A dedicated CT scan of the right thigh showed that the mass involved the entire length of the anterior muscle compartment. Core biopsies, performed to rule out extremity soft tissue sarcoma, revealed minute fragments of fibrous proliferation with mixed inflammatory infiltrate containing plasma cells, CD3^+^/CD5^+^ lymphocytes and CD68^+^ histiocytes, with no evidence of viable tumor cells (**Figures 1B–D** and **Table 1**). Patient received 6 more doses of nivolumab before developing immune-related encephalitis, with no evidence of brain parenchymal or leptomeningeal metastases. This was treated with steroids, intravenous immunoglobulin, and rituximab with improvement. Though restaging studies showed stable disease, he was transitioned to hospice care due to marked deterioration in performance status.

**Table 1:** Description of clinical and pathological characteristics of the three patients.

	Age/Sex	Prior systemic therapies	Type of ICI	Site(s) of hyperprogression	Biopsy site	Immunohistochemistry
Case 1	68/M	Sunitinib, Axitinib, Everolimus, Pazopanib	Nivolumab	Right thigh muscle	Thigh	CD3^+^, CD4^+^, CD5^+^ lymphocytes CD68^+^ histiocytes PAX8^-^, CK AE1/3^-^, S100^-^, Desmin^-^
Case 2	72/M	Sunitinib, Axitinib, Everolimus	Nivolumab	Colon, lung, lymph nodes, liver, adrenal	Colon	CD3^+^, CD4^+^, CD5^-^ lymphocytes CD68^+^ histiocytes PAX8^-^, CD10^-^, Renal Cell^-^
Case 3	70/M	Sunitinib, Everolimus, Axitinib, Sorafenib	Nivolumab	Lymph nodes, liver, adrenal, stomach	Stomach	Not performed

**Figure 2:**
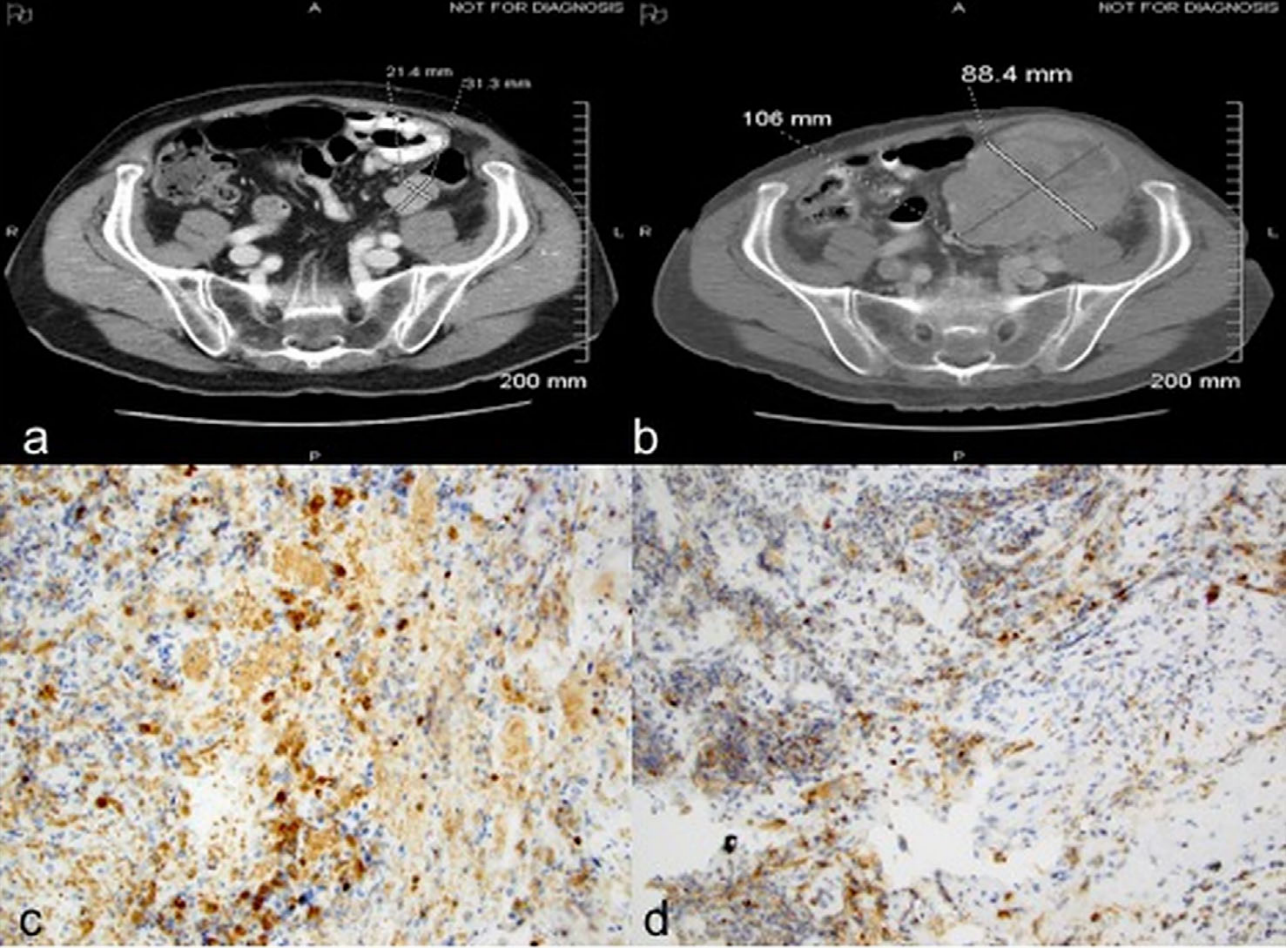
Case 2 demonstrating descending colon mass before **(A)** and after **(B)** nivolumab treatment; **(C)** CD4 positive **(D)** CD 68 positive immunohistochemical staining.

Case 2: A 72-year-old male underwent left radical nephrectomy for a 7.5 cm pT3aNx, Fuhrman grade 3, clear cell RCC. CT scans 9 months later showed enlarging bilateral pulmonary nodules, biopsy of which confirmed metastatic RCC. Over the next two years he was treated with sunitinib, axitinib, and everolimus on various clinical trials. Eventually, due to disease progression, he was initiated on nivolumab 3 mg/kg every 2 weeks. Staging scans at this time revealed multiple bilateral pulmonary nodules, an enlarged left external iliac lymph node, and a small descending colon mass. Follow up CT after 6 doses of nivolumab showed interval worsening of lung metastases, left hilar adenopathy, new liver and right adrenal metastases, and marked enlargement of the descending colon mass from 2.1 x 3.1 cm to 8.8 x 10.6 cm (**Figures 2A, B**). He later developed bloody stools with left lower quadrant abdominal pain. Colonoscopy revealed a large nearly-obstructing mass in the descending colon, biopsies of which showed necrosis, acute and chronic inflammation with fibrin, but no evidence of viable malignancy. Immunohistochemistry revealed CD4^+^ infiltrating lymphocytes and CD68^+^ histiocytes (**Figures 2C, D** and **Table 1**). After recovery from the acute event, he received 3 more treatments with nivolumab but experienced marked decline in performance status before subsequent restaging, and eventually opted for hospice care.

**Figure 3:**
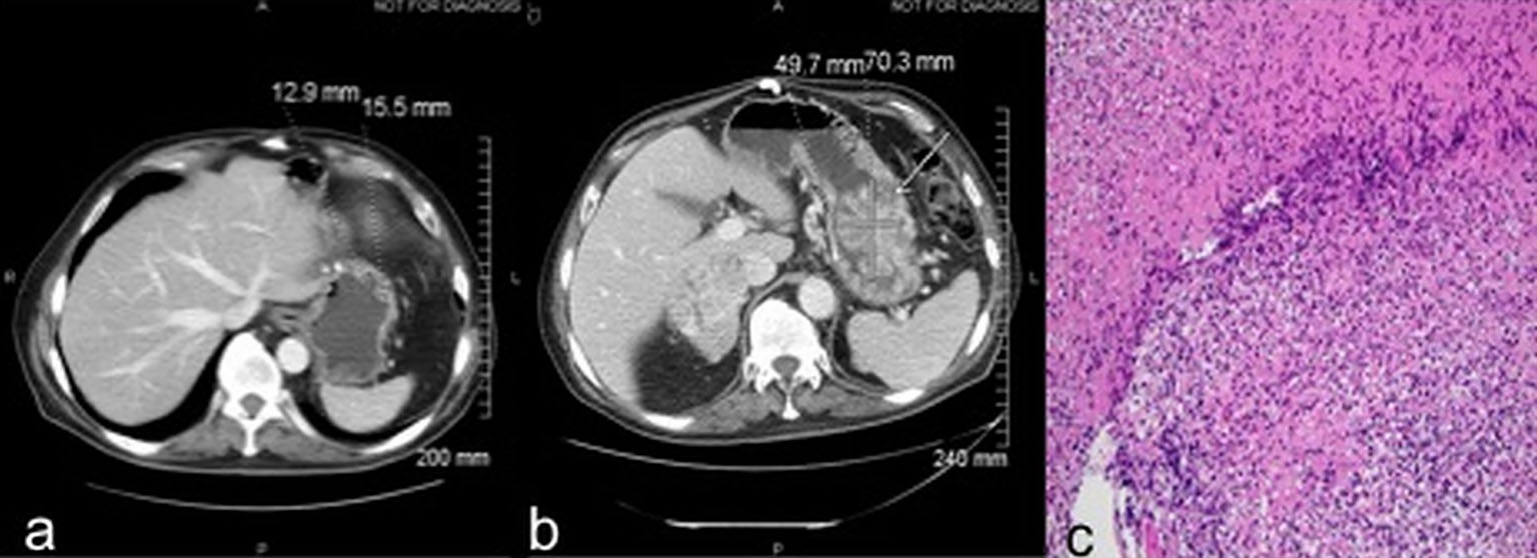
Case 3 demonstrating stomach mass before **(A)** and after **(B)** nivolumab treatment **(C)** extensive necrosis with no viable tumor (H&E, x100).

Case 3: A 70-year-old male presented with abdominal pain and gastrointestinal bleeding and was found to have a large fatty tumor in the gastric body and a bulky lobulated mass in the superior pole of left kidney. He underwent resection of the gastric mass and a left radical nephrectomy. Pathology on the former showed a lipoma while nephrectomy revealed a 7.2 cm pT3aNx, Fuhrman grade 2, clear cell RCC. CT scans three years later revealed a solitary left lower lobe lung nodule, which was biopsied to confirm RCC, and treated with VATS resection. MRI brain 6 months later revealed a large temporal lobe lesion which was resected followed by gamma knife radiosurgery to the resection cavity. He then developed another brain lesion 4 months later and underwent a second gamma knife treatment. Subsequently, he received multiple systemic therapies including sunitinib, everolimus, axitinib, and sorafenib over the subsequent 4.5 years but had interval progression in hepatic, right adrenal and subcarinal lymph node metastases. Nivolumab was then initiated at 3 mg/Kg every 2 weeks. CT scans after 6 doses demonstrated interval increase in mediastinal/hilar lymphadenopathy, right adrenal gland and development of new hypodense liver lesions. There was also marked interval enlargement of a multilobular mass tethered to the gastric wall, from 1.3 x 1.5 cm to 5.0 x 7.0 cm, concerning for metastasis versus a second primary cancer (**Figures 3A, B**). Upper endoscopy demonstrated a large, bulky mass in the proximal stomach, highly suggestive of malignancy. However, biopsies revealed only extensive necrosis and inflammatory changes with focal granulation tissue response, and no evidence of viable malignancy (**Figure 3C**). He then received 6 more doses of nivolumab and follow up imaging showed disease progression within the hepatic, splenic and adrenal metastases as well as further enlargement of the gastric mass, thus nivolumab had to be discontinued. The patient was eventually transitioned to hospice care due to rapidly deteriorating performance status.

## Discussion

PD-1 and other checkpoints are critical to tumor-induced immune evasion, and antibodies against immune checkpoints have emerged as principal tools in the therapeutic arsenal against many cancers (1). While on ICIs, differentiating normal tumor progression from atypical responses, such as pseudoprogression and hyperprogression, can prove to be challenging. Pseudoprogression is characterized by an initial increase in tumor size or appearance of new lesions followed by tumor regression and clinical benefit with continued treatment (4, 6). The underlying pathophysiology includes effector immune-cell infiltration with resultant tumor inflammation and/or interval tumor growth before immune mechanisms are primed to trigger an antitumor response (7–9). The appearance of new lesions may reflect enlargement of preexisting radiographically undetectable metastases due to similar mechanisms. Hyperprogression, on the other hand, is a rarer and more dramatic acceleration in tumor progression after the initiation of ICIs, usually with prompt clinical deterioration (10). Precise mechanisms underlying hyperprogression remain unclear.

Here, we describe three patients with metastatic clear cell RCC who had striking acceleration in tumor growth on cross-sectional imaging after the initiation of nivolumab, associated with detrimental outcomes. Biopsies from sites of radiographic progression revealed extensive areas of necrosis and lymphohistiocytic infiltration with no viable tumor. These changes indicate exuberant antigen presentation, T-cell cytotoxicity, and macrophage-mediated scavenging, findings previously reported in patients with ICI-induced pseudoprogression (7–9). However, our patients had marked acceleration in tumor growth with profound clinical deterioration suggesting rapid loss of response and resulting in transition to hospice.

Tumor histology after hyperprogression has not been well-characterized. One study examining gastric cancer tissue samples before and after anti-PD1 therapy, demonstrated marked increase in tumor-infiltrating proliferative (Ki67^+^) regulatory T-cells (T_regs_) in patients with HPD (n = 2), contrasting with their reduction in those without HPD (n=12) (11). Arasanz et al. (12) described expansion of CD28^-^ CD4^+^ highly differentiated T-cells (T_HD_) in the peripheral blood of NSCLC patients who developed HPD. Another study observed tumor infiltration by M2-like CD163^+^ CD33^+^ PD-L1^+^ macrophages, albeit in pretreatment tissue samples, in NSCLC patients who went on to develop hyperprogression (n = 39) (13). Recently, it was observed that the expansion of intratumoral clonal T cells is associated with a similar proliferation of non-exhausted T cells in the peripheral blood and adjacent non-tumor tissue, suggesting an extratumoral source of tumor-targeted T cells (14). We had previously postulated that patients with metastatic RCC treated with interleukin 2-based immunotherapy respond because of a pre-existing state of immune preparedness (15). Mechanisms underlying rapid tumor progression in RCC after ICI therapy are largely unknown. Our current observations in these three patients support the hypothesis that rapid proliferation of pre-existing T cell clones with exuberant tumor inflammation can result in a cytokine release syndrome-like picture and subsequent clinical deterioration.

ICI-induced hyperprogression is a rare complication in metastatic RCC. Two previous studies reported incidence rates lower than 1% (16, 17). Remarkably, all patients in our study demonstrated a pattern of rapid progression that was either confined to or disproportionately faster in one of the involved anatomic locations. Kobari et al. described three patients with advanced RCC who had rapid radiographic progression confined to a few sites with clinical deterioration after exposure to an ICI, though post-progression biopsies were not analyzed (18). Very little is known about the incidence and mechanisms underlying organ tendencies in hyperprogression across tumor types.

Our report points to a possible discordance between tumor growth kinetics and histology in some patients with rapid radiographic progression while on treatment with ICIs, as demonstrated by the absence of viable cancer on biopsies from sites of such progression. Exuberant antitumor immunity and cytokine release secondary to overwhelming inflammation may contribute to rapidly declining performance status and other detrimental outcomes in these patients (19). Tumor biopsies should be carefully considered in such patients, preferably in the context of well-designed prospective studies, to further characterize these immune responses and identify potential therapeutic targets. We also hypothesize that evaluation of serum cytokine profiles in such cases may inform potential salvage strategies, such as the IL-6 inhibitor tocilizumab (20).

Our study was limited by its retrospective nature and small sample size. We cannot rule-out that sampling bias due to intratumor heterogeneity could have accounted for the absence of viable tumor in biopsy specimens, though multiple cores were examined in these three patients. Since biopsies were performed as part of routine care, primarily to rule out second primary neoplasms, immune-cell subpopulations in the inflammatory infiltrates could not be further characterized, and immunohistochemical staining was performed at the discretion of the pathologist. Baseline biopsies prior to the initiation of ICI, from the sites of subsequent rapid radiographic progression, were not available for comparison. Also, serum cytokine levels were not measured in these patients.

## Conclusions

Overwhelming antitumor immune responses may contribute to detrimental outcomes in some patients with ICI-induced radiographic progression. Tumor biopsies and cytokine analyses, preferably in the context of prospective studies, may help elucidate the pathophysiology underlying these aberrant responses.

## Data Availability Statement

The original contributions presented in the study are included in the article/supplementary material. Further inquiries can be directed to the corresponding author.

## Ethics Statement

The studies involving human participants were reviewed and approved by Roswell Park Comprehensive Cancer Center Institutional Review Board. Informed consent was obtained from the individuals' next of kin as they were deceased. Written informed consent was obtained from the individual(s) for the publication of any potentially identifiable images or data included in this article.

## Author Contributions

DG and RJ compiled the data and wrote the manuscript. SG proposed the concept, data acquisition, interpretation and made substantial edits to the manuscript. SM, GA, BX, CL, LH, MS, KR, and ME made major contributions to data acquisition and interpretation. All authors contributed to the article and approved the submitted version.

## Conflict of Interest

The authors declare that the research was conducted in the absence of any commercial or financial relationships that could be construed as a potential conflict of interest.

## References

[1] Wei SC Duffy CR Allison JP. Fundamental Mechanisms of Immune Checkpoint Blockade Therapy. Cancer Discovery (2018) 8(9):1069–86. 10.1158/2159-8290.CD-18-0367 30115704

[2] Motzer RJ Escudier B George S Hammers HJ Srinivas S Tykodi SS. Nivolumab Versus Everolimus in Patients With Advanced Renal Cell Carcinoma: Updated Results With Long-Term Follow-Up of the Randomized, Open-Label, Phase 3 CheckMate 025 Trial. Cancer (2020) 126(18):4156–67. 10.1002/cncr.33033 PMC8415096 32673417

[3] Wang Q Gao J Wu X. Pseudoprogression and Hyperprogression After Checkpoint Blockade. Int Immunopharmacol (2018) 58:125–35. 10.1016/j.intimp.2018.03.018 29579717

[4] Borcoman E Nandikolla A Long G Goel S Le Tourneau C. Patterns of Response and Progression to Immunotherapy. Am Soc Clin Oncol Educ Book (2018) 38:169–78. 10.1200/EDBK_200643 30231380

[5] George S Motzer RJ Hammers HJ Redman BG Kuzel TM Tykodi SS. Safety and Efficacy of Nivolumab in Patients With Metastatic Renal Cell Carcinoma Treated Beyond Progression: A Subgroup Analysis of a Randomized Clinical Trial. JAMA Oncol (2016) 2(9):1179–86. 10.1001/jamaoncol.2016.0775 PMC5568541 27243803

[6] Ferrara R Matos I. Atypical Patterns of Response and Progression in the Era of Immunotherapy Combinations. Future Oncol (2020) 16(23):1707–13. 10.2217/fon-2020-0186 32687405

[7] Rocha P Hardy-Werbin M Naranjo D Taus Á Rodrigo M Zuccarino F. Cd103+Cd8+ Lymphocytes Characterize the Immune Infiltration in a Case With Pseudoprogression in Squamous Nsclc. J Thorac Oncol (2018) 13(10):e193–6. 10.1016/j.jtho.2018.05.008 29775806

[8] Tabei T Tsuura Y Kobayashi K. Pseudoprogression: A Case of Metastatic Renal Clear Cell Carcinoma Treated With Nivolumab. Pathol Int (2018) 68(11):627–9. 10.1111/pin.12714 30151940

[9] Tanizaki J Hayashi H Kimura M Tanaka K Takeda M Shimizu S. Report of Two Cases of Pseudoprogression in Patients With non-Small Cell Lung Cancer Treated With Nivolumab-Including Histological Analysis of One Case After Tumor Regression. Lung Cancer (2016) 102:44–8. 10.1016/j.lungcan.2016.10.014 27987588

[10] Frelaut M Le Tourneau C Borcoman E. Hyperprogression Under Immunotherapy. Int J Mol Sci (2019) 20(11):2674. 10.3390/ijms20112674 PMC6600249 31151303

[11] Kamada T Togashi Y Tay C Ha D Sasaki A Nakamura Y. Pd-1(+) Regulatory T Cells Amplified by PD-1 Blockade Promote Hyperprogression of Cancer. Proc Natl Acad Sci U S A (2019) 116(20):9999–10008. 10.1073/pnas.1822001116 31028147 PMC6525547

[12] Arasanz H Zuazo M Bocanegra A Gato M Martínez-Aguillo M Morilla I. Early Detection of Hyperprogressive Disease in Non-Small Cell Lung Cancer by Monitoring of Systemic T Cell Dynamics. Cancers (Basel) (2020) 12(2):344. 10.3390/cancers12020344 PMC7073153 32033028

[13] Lo Russo G Moro M Sommariva M Cancila V Boeri M Centonze G. Antibody-Fc/Fcr Interaction on Macrophages as a Mechanism for Hyperprogressive Disease in Non-small Cell Lung Cancer Subsequent to PD-1/PD-L1 Blockade. Clin Cancer Res (2019) 25(3):989–99. 10.1158/1078-0432.CCR-18-1390 30206165

[14] Wu TD Madireddi S de Almeida PE Banchereau R Chen YJ Chitre AS. Peripheral T Cell Expansion Predicts Tumour Infiltration and Clinical Response. Nature (2020) 579(7798):274–8. 10.1038/s41586-020-2056-8 32103181

[15] Schwaab T Schwarzer A Wolf B Crocenzi TS Seigne JD Crosby NA. Clinical and Immunologic Effects of Intranodal Autologous Tumor Lysate-Dendritic Cell Vaccine With Aldesleukin (Interleukin 2) and IFN-{alpha}2a Therapy in Metastatic Renal Cell Carcinoma Patients. Clin Cancer Res (2009) 15(15):4986–92. 10.1158/1078-0432.CCR-08-3240 PMC3775650 19622576

[16] Dionese M Pierantoni F Maruzzo M Bimbatti D Deppieri FM Maran M. Fatal Hyperprogression Induced by Nivolumab in Metastatic Renal Cell Carcinoma With Sarcomatoid Features: A Case Report. Anticancer Drugs (2021) 32(2):222–5. 10.1097/CAD.0000000000000991 32868643

[17] Hwang I Park I Yoon SK Lee JL. Hyperprogressive Disease in Patients With Urothelial Carcinoma or Renal Cell Carcinoma Treated With Pd-1/Pd-L1 Inhibitors. Clin Genitourin Cancer (2020) 18(2):e122–33. 10.1016/j.clgc.2019.09.009 31837940

[18] Kobari Y Kondo T Takagi T Omae K Nakazawa H Tanabe K. Rapid Progressive Disease After Nivolumab Therapy in Three Patients With Metastatic Renal Cell Carcinoma. In Vivo (2017) 31(4):769–71. 10.21873/invivo.11129 PMC5566938 28652455

[19] Slota A Khan R Rahman A Warner EA. Cytokine Release Syndrome As a Rare Complication of Nivolumab: A Case Report. Blood (2019) 134(Supplement_1):5630–0. 10.1182/blood-2019-127586

[20] Shimabukuro-Vornhagen A Godel P Subklewe M Stemmler HJ Schlößer HA Schlaak M. Cytokine Release Syndrome. J Immunother Cancer (2018) 6(1):56. 10.1186/s40425-018-0343-9 29907163 PMC6003181

